# How Much is Universal Accessibility Actually Taught in Canadian Occupational Therapy Programs?

**DOI:** 10.1177/00084174251340647

**Published:** 2025-06-02

**Authors:** Alicia Ruiz-Rodrigo, David Gotti, Ernesto Morales, François Routhier

**Keywords:** Accessibility, Curriculum, Environment, Occupational therapy, Training, Accessibilité, environnement, ergothérapie, formation, programmes d’études

## Abstract

**Background.** The environment is essential to occupational participation. However, the involvement and training of occupational therapists in universal accessibility (UA) seems limited. **Purpose.** To explore the content on UA taught in university occupational therapy programs in Canada. **Method.** This study adopts a mixed methodology. A survey, including mostly quantitative data, was distributed to occupational therapy programs across Canada, and course syllabi related to UA, providing qualitative data, were requested. Analysis included descriptive statistics and content descriptive analysis. **Findings.** Thirteen out of 14 programs responded to the survey and five provided course syllabi. While all programs cover UA content, only seven offer specific courses on accessibility. Internships related to UA are offered by seven programs. Some gaps identified include limited knowledge, lack of in-depth content, and limited interprofessional collaboration. There are variations in emphasis on UA and teaching approaches. Boundaries between universal approaches and knowledge of specific disability groups are poorly delineated. UA is unanimously considered essential in the training of occupational therapists. **Conclusion.** The content in UA is heterogeneous across programs. Consensual definitions of accessibility concepts related to occupational therapy are needed to better define the role of the occupational therapist in this area.

## Résumé

**Description.** L’environnement est essentiel à la participation occupationnelle. Cependant, la participation et la formation des ergothérapeutes en matière d’accessibilité universelle semblent limitées. **But.** Explorer le contenu sur l’accessibilité universelle enseigné dans les programmes universitaires d’ergothérapie au Canada. **Méthodologie.** Cette étude adopte une méthodologie mixte. Un sondage, comprenant principalement des données quantitatives, a été distribué aux responsables de programmes d’ergothérapie partout au Canada, et des plans de cours liés à l’accessibilité universelle, fournissant des données qualitatives, ont été demandés. L’analyse comprenait des statistiques descriptives et une analyse descriptive du contenu. **Résultats.** Les responsables de 13 des 14 programmes ont répondu au sondage, et 5 ont fourni des plans de cours. Alors que tous les programmes offrent du contenu sur le thème de l’accessibilité universelle, seulement 7 offrent des cours portant spécifiquement sur l’accessibilité. Des stages en lien avec l’accessibilité universelle sont offerts dans 7 programmes. Parmi les lacunes relevées, mentionnons les connaissances limitées, le manque de contenu approfondi et la collaboration interprofessionnelle limitée. Les approches pédagogiques et l’accent mis sur l’accessibilité universelle varient. Les frontières entre les approches universelles et les connaissances relatives à certains groupes de personnes en situation de handicap sont mal définies. L’accessibilité universelle est unanimement considérée comme essentielle dans la formation des ergothérapeutes. **Conclusion.** Le contenu sur l’accessibilité universelle est hétérogène d’un programme à l’autre. Des définitions consensuelles des concepts d’accessibilité liés à l’ergothérapie sont nécessaires pour mieux définir le rôle de l’ergothérapeute dans ce domaine.

## Introduction

The occupational therapy profession has undergone a rapid and significant evolution since its foundation (Boyt Schell & Gillen, 2019). Indeed, the unique expertise of occupational therapists is playing an increasingly central role in promoting the participation of people with one or more disabilities (Egan & Restall, 2022a). While participation depends as much on occupation, personal factors, and environmental factors, initial occupational therapy interventions rarely focus on the latter (Christiansen et al., 2014). However, the importance of environmental supportiveness has gradually been recognized as an essential feature for disabled people (Law, 1991) (in this article, both person-first and identity-first language are used, acknowledging that language preferences vary between individuals and communities (American Psychological Association, 2020, Andrews et al., 2022, Dunn & Andrews, 2015)). Indeed, in the 1990s, new conceptual models (both interprofessional and specific to occupational therapy), based on social model, were developed, supporting in particular the development of accessible spaces. These models share the common consideration of the transactional nature of the relationships between the environment, the person and the performance of activities (Morel-Bracq, 2017). In that perspective, professional organizations, such as Quebec Professional Code or the World Federation of Occupational Therapy strongly support the inclusion of environmental support in occupational therapy interventions (Code des professions, 2021; World Federation of Occupational Therapy, 2024).

Similarly, the importance of a supportive environment is widely acknowledged in social and political discourse (Loi canadienne sur l’accessibilité, 2019). Standards, conventions and laws on this subject have been created. For example, in 2006, the United Nations published the Convention on the Rights of Persons with Disabilities (2006). This convention, which includes accessibility among the rights of people with disabilities, has now been ratified by 185 countries. The states that consent to the convention have to include

appropriate measures to ensure to persons with disabilities access, on an equal basis with others, to the physical environment, to transportation, to information and communications, including information and communications technologies and systems, and to other facilities and services open or provided to the public, both in urban and in rural areas. (Convention on the Rights of Persons with Disabilities, 2006)

This is done in order to enable their full participation in all aspects of life. In Canada, the first federal legislation concerning accessibility was enacted in 2019. The objective of this legislation is to make Canada a barrier-free nation by 2040. This encompasses a range of barriers, including those related to the physical or architectural environment, information and communication, behavior and technology, and policy and practice (Loi canadienne sur l’accessibilité, 2019).

Several key concepts have emerged from the growing interest in making environments accessible to diverse populations. Initially, “barrier-free design” was narrowly associated with wheelchair accessibility (Persson et al., 2015; Steinfeld & Maisel, 2012). This notion has evolved into broader terms such as “universal accessibility” (UA), to take into account the diversity of possible users. UA is defined as “the character of a product, process, service, environment, or information that, with the aim of equity and in an inclusive approach, enables any person to carry out activities independently and to obtain equivalent results” (Rocque et al., 2011). Related terms have emerged, including “design for all”, “inclusive design” and “universal design” (Dolph, 2021; Persson et al., 2015). While these terms focus on the design process, the term “accessibility” describes the actual usability of an environment by diverse populations. Evidently, many existing environments have not been designed with UA in mind. Therefore, this study uses the term “accessibility” to recognize that while universal design is ideal for new environments, existing spaces often require modifications to become more accessible to diverse populations. Core to this evolution is the concept of “universality”—the idea that environments should be suitable for the widest possible range of users.

The notion of “universality” has already been contested in literature for its utopian nature (Fougeyrollas et al., 2019). The cultural components of design, the complexity of disability, and its intersectional manifestations collectively make the creation of a universally accessible environment a daunting task (Fougeyrollas et al., 2019). Moreover, the concept of universal design principles potentially incorporates vestiges of the medical model (Imrie, 2012). The limitations of a framework that may prove to be too constricting for the heterogeneity of the population have also been discussed (Fougeyrollas et al., 2019; Imrie, 2012). According to Imrie (2012), the vision of universal design as a tool for applying design technologies risks omitting important socio-political issues and favoring reductionist interventions. In this regard, the integration of cross-disciplinary teams, and particularly occupational therapists, has the potential to foster a more inclusive and person-centered approach (Lid, 2014) and to address accessibility issues from a macro perspective (Egilson & Jónasdóttir, 2023).

As proposed by Young et al. (2019), the role of the occupational therapist in the domain of UA is to facilitate the creation of environments that enhance functional capabilities and, in turn, encourage user engagement. The common ground between UA and occupational therapy is their foundation in a person-centered approach (Steinfeld & Maisel, 2012; Young et al., 2019). However, with their vision centered on the uniqueness of the person, occupational therapists can prevent the potential pitfalls of a disability-centered approach. Indeed, it is no longer sufficient to adopt a linear perspective on the personal or contextual factors that can lead to a situation of disability (Egan & Restall, 2022b; Fougeyrollas et al., 2018). Rather, environmental access must be considered from a transactional and dynamic perspective, considering the interactions between individuals, their aspirations, and the contexts in which they engage in activities (Egan & Restall, 2022b; Hamraie, 2012). Occupational therapists possess a unique vantage point, well-suited to challenging the conventional “normal” person model (typically represented by a young, fit male) that often informs design (Hamraie, 2012). Instead, they advocate for a nuanced understanding of human diversity, encompassing individuals with varying attributes and abilities. From this perspective, occupational therapists offer a unique contribution to the field of UA. They are able to utilize their distinctive and comprehensive understanding of occupation to emphasize crucial elements that should be intrinsic to accessible environments for a diverse population.

Despite the recognition of the importance of environmental supportiveness and UA, people with disabilities, which represent 27% of people aged 15 and over in Canada (Statistique Canada, 2022), continue to experience accessibility challenges in the community. Indeed, according to the Canadian Survey on Disability, 72% of Canadians with disabilities reported encountering accessibility barriers over the past year. Notably, 60% of these individuals experienced difficulties navigating indoor and outdoor public spaces due to accessibility barriers (Statistics Canada, 2023). The high prevalence of accessibility issues faced by disabled people in Canada underscores the need for OTs to expand their role and competencies in UA. However, few studies clearly define the role of occupational therapists in UA (Young et al., 2019). To better understand and define the role of the occupational therapist in the field of UA, we first need to understand the training and tools provided in OT programs.

The objective of this study is therefore to explore the content on UA taught in university OT programs in Canada. By investigating how UA is integrated into the education of occupational therapists, we can better understand the preparedness of future occupational therapists in the promotion of inclusive practices in public spaces.

## Methods

For this study a mixed design (Creswell & Plano Clark, 2018) was adopted. The quantitative portion comprised an online survey sent to all university occupational therapy programs in Canada. The qualitative portion consisted of analyzing course syllabi for UA related courses available in these programs. A mixed method was chosen to gain a more comprehensive understanding of the research issue. In addition to the place and scope of UA content in courses, we also wanted to understand the gaps and nuances, as perceived by instructors. In this specific case, the instructors have experience with the content taught and the associated issues. Furthermore, triangulation of results using multiple methods increases the accuracy and credibility of interpretations (Braun & Clarke, 2021; Williams & Morrow, 2009).

### Participants and Data Collection

All occupational therapy programs at Canadian universities were included in the data collection process. The directors of the 14 programs were contacted via email. Contacts were identified using information available on the Association of Canadian Occupational Therapy University Programs website (Association of Canadian Occupational Therapy University Programs, 2022). The directors were requested to identify the individual best suited in their department to respond to the survey, for example, the individual responsible for the UA course, if one existed. Alternatively, they were invited to respond themselves, particularly in instances where no dedicated accessibility course was available.

The data were collected via an online survey developed on the Lime Survey platform (Limesurvey GmbH, 2003) which was distributed via email to potential participants. The survey consisted of 24 questions, divided into three sections, which aimed to provide a descriptive portrait of the UA content in OT programs and its place within each program. The first section of the survey inquired about the respondent's characteristics, as well as the occupational therapy program with which they were affiliated and contributed to the contextualization of the data. The second section included questions regarding the characteristics of any specific UA course offered within the program, if available, as well as other theoretical training opportunities, such as UA content integrated into other courses or the option to enroll in courses from other programs. Additionally, the section explored the availability of practical experience, including internships, and identified potential gaps in UA training within the curriculum. The third section focused on respondents’ perception of the importance of UA in occupational therapy. Furthermore, the survey included a question that enabled respondents from programs that offered a course in UA to submit their course outlines. Due to the lack of consensus in the literature regarding accessibility terminology, UA was the only term used in the survey, and a definition of this term was included several times. The survey questions were developed by the research team, including experts in rehabilitation and accessibility, with consideration of the existing literature on occupational therapy curricula and the role of occupational therapists in accessibility (e.g., Gomes & Emmel, 2020; Simmons et al., 2010; Wittich et al., 2017). In the process of developing, the input of an occupational therapist with a specialization in UA was sought in order to validate the relevance and comprehensibility of the questions. The survey was available in both English and French. For illustrative purposes, sample questions are provided in Table 1.

**Table 1:** Examples of Questions in the Online Survey.

Section	# Question	Question sample
Respondent and program information	5	Are you responsible for a course with UA content?
UA content in the Occupational Therapy program	9	Is there a specific course in your occupational therapy curriculum on UA?
UA content in the Occupational Therapy program	21	Is it possible for a student to complete an internship in a facility specializing in UA? (e.g., architectural firm, city department)
Perception of the importance of UA	24	In your opinion, on a scale of 0–10, how important is it to address UA in a professional occupational therapy education program? (10 being the most important and 0 being unimportant)

### Data Analysis

The data obtained from the online survey were subjected to analysis using descriptive statistics (median, range, frequencies) in order to present a comprehensive picture of the UA of content across all occupational therapy programs. Concurrently, content descriptive qualitative analyses (Jiggins Colorafi & Evans, 2016; Sandelowski, 2000) were conducted on the course syllabi obtained. To this end, two individuals (ARR and DG) developed the analysis (Miles et al., 2014; Saldaña, 2013) of the course outlines (*n* = 5) in accordance with the following procedure. First, a course outline was jointly analyzed to identify the content and determine the codes covering that content. This first step was validated with all authors during a team meeting. Next, each person analyzed two additional syllabi individually, and the results were discussed as a team, including all the authors of the article. Three meetings were required to develop, refine and validate the analysis codes.

The study was approved by the sectorial ethics committee on research in rehabilitation and social integration of the *Centre intégré universitaire de santé et de services sociaux de la Capitale-Nationale* (#2022-2422).

## Results

The results of this study are presented in two sections: (1) results from the analysis of data collected via the online survey and (2) results from the analysis of collected syllabi.

### Online Survey

The data have been compiled and presented in a manner that ensures the anonymization of each program or university. Of the 14 university occupational therapy programs, 13 (92.9%) responded to the survey. The respondents were heterogeneous in terms of age (between 47 and 65 years old), gender, position, and status with regard to responsibility for an UA course. The characteristics of the participants are shown in Table 2.

**Table 2:** Participants’ Characteristics (*n* = 13).

	*n*	%
*Age*		
40–50	3	23.1
51–60	8	61.5
61–70	1	7.7
Did not provide age	1	7.7
*Gender*		
Woman	9	69.2
Man	4	30.8
Non-binary	0	0
*Position*		
Head of the program	2	15.4
Full professor	4	30.8
Associate professor	4	30.8
Assistant professor	1	7.7
Clinical faculty lecturer	2	15.4
*Responsible of UA course*		
Yes	7	53.9
No	6	46.1

While all programs report including content on UA, the context in which it is addressed varies considerably. Seven programs indicate that they have a specific course on UA. Six of these programs also offer content on the subject as part of other courses within the program. The remaining six programs have no dedicated courses on UA, but report having content on this topic distributed across several courses within the program (see Table 3).

**Table 3:** Universal Accessibility Content (*n* = 13).

	Specific course on UA					
	Yes (*n* = 6)		No (*n* = 7)		Total (*n* = 13)	
	*n*	%	*n*	%	*n*	%
*Content in other courses*						
Yes	5	38.5	7	53.9	12	92.3
No	1	7.7	0	0.0	1	7.7
*Mandatory/optional*						
Mandatory	5	38.5	4	30.8	9	69.2
Optional	1	7.7	1	7.7	2	15.4
No answer	0	0.0	2	15.4	2	15.4
*Other course at the same time*						
Yes	1	7.7	1	7.7	2	15.4
No	0	0.0	2	15.4	2	15.4
No answer	5	38.5	4	30.8	9	69.2
*Available each year*						
Yes	6	46.1	5	38.5	11	84.6
No	0	0.0	2	15.4	2	15.4
*Possible internship in UA*						
Yes	3	23.1	4	30.8	7	53.9
No	3	23.1	3	23.1	6	46.1

In regard to the presentation of UA content, the context and modalities vary across programs, even in the absence of a dedicated UA course. Firstly, in the majority of programs (*n* = 9), UA content is presented as part of mandatory courses. Furthermore, in two of the four programs that offer UA content in optional courses, other courses are concurrently provided. In recent years, the number of students enrolled in these elective courses has ranged from one to ten. In one program, the enrollment has ranged from one to six, while in the other, it has reached ten students per year. The content is available every year in 11 out of 13 programs (84.6%), while two programs do not offer the content on an annual basis.

It is possible to gain insight into the concept of UA through the utilization of alternative pedagogical techniques, such as enrolling in supplementary courses or undertaking internships. Access to courses in other programs is permitted in only one program (7.7%). Nevertheless, seven programs (53.9%) provide internship opportunities in settings related to UA, occasionally as part of a role-emerging or innovative internship. For instance, students may engage in community-based initiatives that facilitate the inclusion of individuals with disabilities in local communities. These may include placements in municipal departments, community organizations, or the Office for Students with Disabilities at the university.

The respondents identified several gaps in the teaching of UA in their programs. These included a lack of in-depth or advanced knowledge, a lack of cross-sector collaboration with other professionals, a lack of mandatory content, a lack of content on UA for diverse populations, a lack of recognition of courses external to the program, and a lack of consistency and conceptual coherence in the teaching of this topic.

**Figure 1.**
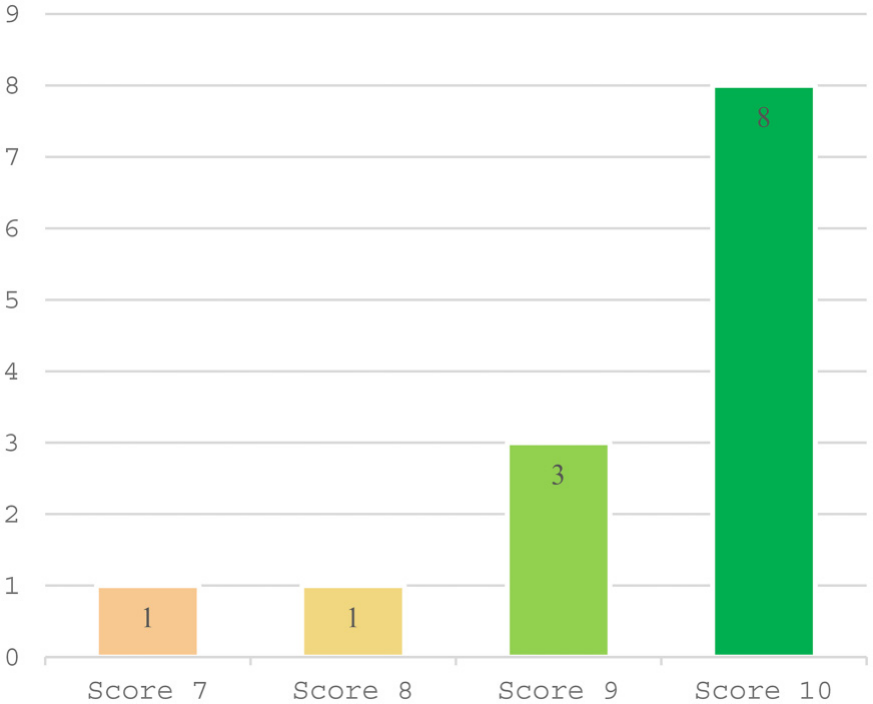
Importance of Universal Accessibility.

Finally, respondents evaluated the importance of incorporating UA into occupational therapy programs on a scale of 1‒10. The ratings ranged from 7 to 10 out of 10, with 61.5% of respondents indicating a rating of 10 out of 10, 23.1% indicating a rating of 9 out of 10, and 7.7% indicating a rating of 7 and 8 (Figure 1).

### 2. Courses Syllabi

In terms of the content of the courses, five programs (38.7%) provided course outlines that included material related to UA, while only two of these (15.4%) indicated that they had a dedicated course on UA. The number of credits assigned to these courses varies, with a range of 1–3 credits. However, the number of credits indicated in the course outlines refers to the entire course, rather than solely to the concepts related to UA, for which the time allocated is also variable. The terms “universal accessibility” and “universal design” are present in all course outlines, yet they are not defined within the document. Rather, defining these concepts would be one of the objectives of some of the teaching activities.

Several of the courses address UA within the context of a continuum of occupational therapy interventions. Firstly, the occupational therapist’s role is situated in terms of intervention in the environment, and in relation to the roles of other professionals. Indeed, a considerable number of programs acknowledge the significance of intersectoral collaboration in environmental intervention. While the persons responsible for the courses appear to possess a particular expertise in occupational therapy, the people who collaborate in teaching these courses often have a variety of expertise (e.g., architecture, industrial design, or speech therapy). However, activities involving students or professionals from other disciplines appear to be limited to isolated instances.

The evaluation of the environment is presented through approaches that prioritize the appreciation of the individual's occupational participation in that environment. The assessment process is supported through the teaching of specific concepts and strategies pertinent to specific disabilities. For instance, some courses examine the principles of individual and personalized assistive technologies or environment modifications, which are not directly related with UA or universal design.

Finally, many key concepts are presented with the objective of equipping students to act on the environment during their interventions. Indeed, several concepts are designed to facilitate students’ reasoning processes in the development of interventions. Students integrate notions of ergonomics, universal design, and biomechanics to develop intervention modalities. The curricula encompass a multitude of themes that propose tangible methods for facilitating individuals’ engagement in their environment. These include assistive technologies, strategies for modifying the environment, specialized programs and services within the community, and other pertinent considerations. In the majority of cases, the objective of the intervention process is to enable disabled people to engage in occupational activities within the community, but it does not focus on the development of accessible environments.

Most courses specify the applicability of concepts to groups in their general course description. However, presentations, cases and clinical vignettes often focus on specific profiles of people with disabilities (e.g., wheelchair users). While the course description suggests a generalization of the concepts to diverse and broader groups, it is not possible to be certain of this on the basis of the content of the course outlines.

In terms of assessment and teaching methods, all courses include mandatory readings, lectures, conferences and case studies to support theoretical learning of key concepts. They also incorporate critical group discussions focused on specific cases. While common strategies are used, there are also different and varied modalities between institutions. In two courses in particular, a mixed method combining lectures and laboratories is used. Students observe and use technological aids and environmental adaptations to develop their clinical reasoning. For these same courses, visits to living environments for people with disabilities enable students to apply their knowledge in a real-life context. One of these courses also incorporates self-study activities. All courses use individual and group assessment methods. Individual exams are used to assess students’ knowledge of theoretical concepts covered in presentations and laboratories. Team-based assessments of clinical skills, in the form of video content creation on target themes, applied projects or oral presentations, are also used.

## Discussion

This study examined the UA content taught in university occupational therapy programs in Canada. The findings shed light on the heterogeneity and discrepancies in pedagogical approaches and definition of UA between programs at different universities. The number of hours devoted to the subject, the manner in which the content is taught, and the content itself vary. The methodology used, particularly the analysis of course syllabi, made it possible to observe these differences. This heterogeneity raises questions about the potential impact on students’ perceptions of the role of the occupational therapist in the field of UA and environmental design. However, this study provides important insights into the conceptual gaps in UA, the need of collaboration between professional from different disciplines (cross-sector initiatives), the skills that students are developing, and the importance of specialized courses in the education of occupational therapists.

Despite the lack of consistency between programs, the fact that all of them include content on UA shows some interest in this topic. Furthermore, the high scores given by respondents when asked about the importance of this issue reflect an acceptance of the role of the occupational therapist in this area.

However, the possibilities offered by some of the programs are limited because the content is offered in elective courses or is spread over courses that are not specific to the subject and diluted in other concepts that are more or less related to accessibility (e.g., task analysis and assistive technology). This may limit both, the interest that students can develop in the field, and the acquisition of in-depth knowledge and overall understanding of the field. Bar and Ratzon (2016) showed that occupational therapy students’ basic knowledge of accessibility increased after a brief training on the topic as part of another course in the program. However, students’ interest and awareness in this area did not increase after the training. Although increasing student interest in a given topic usually seems to be a challenge, some potential solutions could also fill some of the gaps identified by respondents of this study. For example, it would be important to add more comprehensive and coherent training strategies, such as direct involvement of people with disabilities (Bar & Ratzon, 2016; Egilson & Jónasdóttir, 2023) or cross-sector collaboration.

### The Diversity of Understandings and Applications of Universal Accessibility

First, there is a disparity in the understanding and application of the concept of UA and the diversity of terms used in courses syllabi, which coincides with the lack of consensus on terminology in the literature (Dolph, 2021; Persson et al., 2015). This diversity may influence not only the teaching of the concept but also its importance in curriculum. Perspectives on UA are heterogeneous, and few specifically address its application in occupational therapy and the roles and competences of occupational therapist in this field. This observation resonates with the conceptual and implementation gaps associated with UA in occupational therapy (Gomes & Emmel, 2020; Hansen et al., 2021). This situation presents teaching challenges that may hinder the integration of UA into the future practice of occupational therapy students.

While there is a body of literature presenting the concepts of UA, few focus on their application by occupational therapists (Young et al., 2019). Nevertheless, the World Federation of Occupational Therapy (2024) is working to include interventions based on environment accessibility within the occupational therapist's scope of practice.

The concept of UA is not translated within the occupational therapist's mandate: the definitions used in courses are rooted in other disciplines (design, architecture). The understanding, interpretation and implementation of universal design principles in occupational therapy is therefore left in the hands of those who teach them, and ultimately those who apply them. This issue is exacerbated by the heterogeneous context in which the concept is taught. To support teaching, approaches grounded in UA should be further delineated and defined in the specific context of occupational therapy practice. A common definition of the meaning of UA in occupational therapy is a crucial step towards identifying and reducing gaps in its teaching. Next, a precise delineation of the occupational therapist's role and responsibilities in the field of UA is essential. Moreover, the skills required for the application of UA must be linked to the occupational therapy competency framework (Association of Canadian Occupational Therapy Regulatory Organizations et al., 2021), which is often at the root of the objectives pursued by occupational therapy courses. To this end, Egilson and Jónasdóttir (2023) critically reflect on the place of UA in supporting several occupational therapy missions and values (justice and social inclusion).

In addition to its limited translation to occupational therapy, the concept of UA continues to evolve (Fougeyrollas et al., 2019). Experts from many disciplines are developing new tools and technologies to universally support the participation of disabled people through meaningful access. The Rick Hansen Foundation, for example, is developing accessibility ratings that are periodically reviewed to ensure they meet society's needs (Rick Hansen Foundation, 2022). Although this innovation demonstrates enthusiasm for inclusion, integrating the latest practices remains a time-consuming and resource-intensive challenge for professors.

### Cross-Sector Initiatives

Even if different professionals are involved in some of the courses related to UA, the lack of cross-sector collaboration in accessibility teaching was one of the gaps identified by respondents of this study. To refine the understanding of UA, especially in occupational therapy, several authors stress the importance of cross-sector initiatives (Lid, 2014). In particular, collaboration with individuals from social work or sociology backgrounds enables a better understanding of the role of society in people's participation, or with architects and designer who also have a responsibility in developing an inclusive society (Lid, 2014). Although accessibility is a characteristic of the environment, the concept of accessibility is understood in the context of person–environment interaction (Iwarsson & Stahl, 2003). Mobilizing different expertise can help to make the real issues visible on a societal scale and identify means of intervention.

This position echoes the desire of respondents in our study to integrate more cross-sector collaboration into the curriculum. Occupational therapists have a fundamental role to play in the field of UA because of their knowledge of the individual and the person-environment interaction that takes place in occupations. However, in the field of UA research, occupation seems to be the least addressed element in relation to the person and the environment (Watchorn et al., 2021). Indeed, (Egan & Restall, 2022b) warn of the risks of underestimating the complexity of occupations and the context in which they are undertaken. The broader the scope of occupational therapy interventions, the more numerous and complex the contextual influencing factors. In addition, although the programs included in this study address several individual profiles in terms of accessibility, it is not possible to ensure exhaustive representation of all population groups that could benefit from UA. It is therefore essential to collaborate and integrate the perspectives of other professionals, such as architects, engineers or design experts, in order to promote a more holistic vision of the key issues in the social context. Ultimately, these collaborations help to highlight hidden barriers in social systems and public spaces (Egan & Restall, 2022b).

### Specific Reasoning and Challenges in Applying Universal Accessibility

In addition to conceptual issues, the application of UA is challenging because of the specific skills and therefore a shift in clinical reasoning is required. On the one hand, the courses included in the analysis demonstrate the tension between teaching approaches focused on reducing environmental barriers and preparing students to intervene with specific groups or clients.

On the other hand, approaches based on UA require a shift in thinking from the individual to a community or even a society and consider all three levels of the environment (micro, meso, and macro) (Egilson & Jónasdóttir, 2023). Learning this mode of clinical reasoning is a major challenge for students, given the lack of mandatory and specialized educational content in UA. As a result, new graduates may feel inadequately prepared to implement universal approaches in their clinical practice (Cowen et al., 2024; Kantartzis & Molineux, 2017).

While it is essential for future occupational therapists to be able to identify issues common to groups, universal access requires a different line of reasoning. Indeed, a universal access approach based on occupational participation implies that biomedical or individual dimensions are not the main focus of clinical reasoning (Egan & Restall, 2022b). It would therefore not be sufficient or representative of the diversity of individuals to consider interventions for a few specific groups selected on the basis of their disability profile. This duality in reasoning supports the importance for occupational therapy programs to offer specific courses in UA. Students should have the opportunity to focus their reasoning on the context in which occupations are performed and develop interventions based on UA approaches rather than on the nature of disability.

Occupational therapy and UA are both founded in a person-centered approach (Young et al., 2019) and many of the key skills acquired by students during their training, such as competencies related to culture, equity and justice domain (Association of Canadian Occupational Therapy Regulatory Organizations et al., 2021) can be transferred to the application of UA in public spaces. Occupational therapists are guided by their professional perspective to acquire expert skills in flexibility, adaptability, critical thinking, and problem solving. These characteristics, which underpin the competency requirements (Association of Canadian Occupational Therapy Regulatory Organizations et al., 2021) for occupational therapists, are central to reasoning in UA, where compromises are often required to meet the diverse, even conflicting, needs of multiple populations. University programs can therefore support students in reflecting on their transversal competencies to prepare them to defend their relevance in emerging roles, particularly in UA.

This situation limits the emergence of new and innovative practice roles in UA. However, the opportunity that some universities offer for emerging internships in settings related to UA could partially fill these gaps. In addition, offering different learning modalities would promote the acquisition of skills by a wider range of students (Simmons et al., 2010).

### The Importance of Universal Accessibility

As mentioned before, the occupational therapy profession is relatively young and has evolved rapidly over the last decades (Boyt Schell & Gillen, 2019). The introduction of UA within the university programmes is a clear manifestation of this evolution. In fact, survey respondents rated the inclusion of content related to UA in occupational therapy programs as very important, giving high marks (between 7 and 10 out of 10) to the related question. Although some of the respondents are responsible for a course on the subject, these results indicate a homogeneous opinion on the matter, reinforcing the importance of including UA content in programs. However, if the profession is to continue to move in the innovative direction in which it has since its implementation, it is necessary to seek certain definitions regarding the role of the occupational therapist and the UA, as well as a definition of the UA concept itself, in order to speak the same language in all university occupational therapy programs in Canada. For occupational therapists, overcoming these challenges is worth the potential of UA for the inclusion of people with disabilities.

### Study Limitations

There are limitations to this study. The data associated with the course syllabi provide an overview of the pedagogical content and are an important contribution to this study, but they do not provide an exhaustive look at the nature of the courses.

On one hand, of all the programs studied, only five institutions shared their course outlines. This issue of access to data limits the ability to make comparisons across institutions. Secondly, the level of detail of the information provided in the documents varies. A consultation of the specific content of each pedagogical modality would be necessary to further clarify the conceptual gaps in the topics taught. On the other hand, the online questionnaire used in this study was designed to minimize the burden on respondents. To this end, because course outlines were requested, some information likely to be found in the outlines was not explored in the questionnaire. Because only five institutions shared course outlines, some data regarding content within each program and course structure are limited.

Concerning the terminology related to accessibility, although only one term (UA) was used and defined in the survey, the lack of consensus in the literature regarding preferred terms and definitions may have been confusing and influenced participants’ responses.

Finally, this study provides a descriptive portrait from the perspective of certain individuals involved in occupational therapy programs. Further studies that include a more in-depth perspective, as well as the opinions of other stakeholders such as clinician occupational therapists with expertise in UA, and particularly disabled people, are needed to understand the gaps and needs in the education of occupational therapists in the UA.

## Conclusion

This study illustrates the heterogeneity in the understanding of UA and the differences in UA curricula among occupational therapy programs in Canada. The lack of specific courses dedicated exclusively to UA makes it difficult to assess the place of this concept in occupational therapy education. UA is rarely treated as a major topic. Rather, the concept is used to support other topics. For example, universal design principles and UA approaches are presented to support pragmatic clinical reasoning for interventions with specific population groups (e.g., the older or obese people). Thus, while some courses focus on UA, it is difficult to assess the place of these theoretical foundations in the occupational therapy process taught in these courses. Given the differences between programs and the fact that content is spread across a number of non-specific courses, other, more in-depth analyses are relevant. For example, a holistic audit of UA content, including all courses in the program, or student evaluations (both of specific courses and of content in the program as a whole) would be avenues to explore in order to identify gaps in content delivery as well as the needs of each program and the profession.

## Key Messages

- Occupational therapy programs include content on UA either in courses considered specific to that topic or spread across program courses, the content and modalities are heterogeneous across programs, and the definition of UA as it relates to occupational therapy is limited.
- Some programs offer innovative initiatives such as emerging role internship opportunities in specific UA settings, which can make a significant contribution to the development of the role of the occupational therapist in this area.
- While there are gaps in UA courses, such as a lack of initiatives that include professionals or students from different disciplines, educators consider UA as a very important component of occupational therapy programs.

## Supplemental Material

sj-pdf-1-cjo-10.1177_00084174251340647 - Supplemental material for How much is Universal Accessibility Actually Taught in Canadian Occupational Therapy Programs? Supplemental material, sj-pdf-1-cjo-10.1177_00084174251340647 for How much is Universal Accessibility Actually Taught in Canadian Occupational Therapy Programs? by Alicia Ruiz-Rodrigo, David Gotti, Ernesto Morales and François Routhier in Canadian Journal of Occupational Therapy
