# Erratum to “How much is Universal Accessibility Actually Taught in Canadian Occupational Therapy Programs? Dans quelle mesure l’accessibilité universelle est-elle enseignée dans les programmes canadiens d’ergothérapie?”

**DOI:** 10.1177/00084174251361650

**Published:** 2025-07-28

Ruiz-Rodrigo A., Gotti D., Morales E., Routhier F. (2025). How much is Universal Accessibility Actually Taught in Canadian Occupational Therapy Programs? Dans quelle mesure l’accessibilité universelle est-elle enseignée dans les programmes canadiens d’ergothérapie? *Canadian Journal of Occupational Therapy*, 0(0). https://doi.org/10.1177/00084174251340647

In this article, the funding details were inadvertently omitted in the OnlineFirst version. The online version has been updated to include the funding information. The corrected funding statement is provided below.

**Funding:** The authors disclosed receipt of the following financial support for the research, authorship, and/or publication of this article: Some members of the research team are currently funded by the Fonds de recherche du Québec: FR as a Senior research fellow of the Fonds de recherche du Québec - Santé (Health) (296761), and ARR et DG as a Doctoral Research Scholarship of the Fonds de recherche du Québec - Société et Culture (Society and Culture) (ARR : 315806; DG : 348335). Université Laval and the Centre interdisciplinaire de recherche en réadaptation et intégration sociale also contributed to the funding of student authors of this project.

