# Supplementary material for: How Much is Universal Accessibility Actually Taught in Canadian Occupational Therapy Programs?
Source: Can J Occup Ther. 2025 Jun 2;93(3):284–93. doi: 10.1177/00084174251340647 (PMC13400796; doi:10.1177/00084174251340647)
Supplement: sj-pdf-1-cjo-10.1177_00084174251340647 - Supplemental material for How much is Universal Accessibility Actually Taught in Canadian Occupational Therapy Programs? [file sj-pdf-1-cjo-10.1177_00084174251340647.pdf]

Universal accessibility training offered in occupational therapy programs

We are conducting a study on **the role of occupational therapy in universal accessibility**. This survey is an analysis of the accessibility training offered to occupational therapy students in Canada.

The survey questions address universal accessibility content in university occupational therapy programs and your responses will help us to develop a picture of this content.

For the purposes of this survey, "universal accessibility" is defined as: the character of a product, process, service, information, or environment that, in an equitable and inclusive manner, enables all persons to perform activities independently and to achieve equivalent results.

The survey should take approximately 20 minutes to complete, and your responses will be kept confidential.

If you have any questions about the survey or the project, please contact us at:

[redacted]

Thank you for your participation!

There are 29 questions in this survey

Respondent and program information

What gender do you identify with?

Please choose **only one** of the following:

- ○ Woman
- ○ Man
- ○ Non-binary
- ○ Other
- ○ I prefer not to answer

How old are you?

Please choose **only one** of the following:

- 18
- 19
- 20
- 21
- 22
- 23
- 24
- 25
- 26
- 27
- 28
- 29
- 30
- 31
- 32
- 33
- 34
- 35
- 36
- 37
- 38
- 39
- 40
- 41
- 42
- 43
- 44
- 45
- 46
- 47
- 48
- 49
- 50
- 51

- 52
- 53
- 54
- 55
- 56
- 57
- 58
- 59
- 60
- 61
- 62
- 63
- 64
- 65
- 66
- 67
- 68
- 69
- 70
- 71
- 72
- 73
- 74
- 75
- 76
- 77
- 78
- 79
- 80
- 81
- 82
- 83
- 84
- 85
- 86
- 87

- 88
- 89
- 90
- 91
- 92
- 93
- 94
- 95
- 96
- 97
- 98
- 99

What is your position?

Please choose **only one** of the following:

- Program Direction
- Full Professor
- Associate Professor
- Assistant Professor
- Faculty lecturer
- Clinical faculty lecturer
- Other

How long have you been in your position?

Please choose **only one** of the following:

- Less than one year
- 1 to 5 years
- 6 to 10 years
- More than 10 years

Are you responsible for a course with universal accessibility content?

Please choose **only one** of the following:

- ◯ Yes
- ◯ No

For the purposes of this survey, "universal accessibility" is defined as: the character of a product, process, service, information, or environment that, in an equitable and inclusive manner, enables all persons to perform activities independently and to achieve equivalent results.

What is your main area of expertise?

Please choose **all** that apply:

- ☐ Occupational Therapy
- ☐ Architecture
- ☐ Engineering
- ☐ Other: ____________

University name

Please choose **only one** of the following:

- ◯ Dalhousie University
- ◯ McMaster University
- ◯ McGill University
- ◯ Queen's University
- ◯ Université Laval
- ◯ Université de Montréal
- ◯ Université de Québec à Trois Rivières
- ◯ Université de Sherbrooke
- ◯ University of Alberta
- ◯ University of British Columbia
- ◯ University of Manitoba
- ◯ University of Ottawa
- ◯ University of Toronto
- ◯ Western University

Number of new students per year in occupationnal therapy program

Only numbers may be entered in this field.

Please write your answer here:

Universal accessibility content in the Occupational Therapy program

For the purposes of this survey, "**universal accessibility**" is defined as: the character of a product, process, service, information, or environment that, in an equitable and inclusive manner, enables all persons to perform activities independently and to achieve equivalent results.

Is there a specific course in your occupational therapy curriculum on universal accessibility?

Please choose **only one** of the following:

- Yes
- No

Comments

Please write your answer here:

Isit exclusive to occupational therapy students?

Please choose **only one** of the following:

- Yes
- No

Comments

Please write your answer here:

Is it mandatory or optional ?

Please choose **only one** of the following:

- Mandatory
- Optional
- Other

Is it offered on the same day and in the same time slot as another optional course?

Only answer this question if the following conditions are met:
Answer was NOT 'Mandatory' at question '13 [Cours3]' (Is it mandatory or optional ?)

Please choose **only one** of the following:

- Yes
- No

When was it last taught?

Please choose **only one** of the following:

- 2022
- 2021
- 2020
- 2019
- 2018
- Avant 2018

Is it still taught each year?

Please choose **only one** of the following:

- Yes
- No

How many students have enrolled in the last three years?

Only answer this question if the following conditions are met:
Answer was NOT 'Mandatory' at question '13 [Cours3]' (Is it mandatory or optional ?)

Please write your answer(s) here:

2021-2022	
2020-2021	
2019-2020	

To your knowledge, is there any content related to universal accessibility integrated into other courses in your occupational therapy program?

Please choose **only one** of the following:

- Yes
- No

In which course(s)?

Only answer this question if the following conditions are met:
Answer was 'Yes' at question '18 [Cours8]' (To your knowledge, is there any content related to universal accessibility integrated into other courses in your occupational therapy program?)

Please write your answer(s) here:

How much time (hours) is devoted to it?

Only answer this question if the following conditions are met:
Answer was 'Yes' at question '18 [Cours8]' (To your knowledge, is there any content related to universal accessibility integrated into other courses in your occupational therapy program?)

Please write your answer here:

Comments

Please write your answer here:

Do students in your occupational therapy program have the opportunity to enroll in a universal accessibility course in another program (e.g., architecture) in your university?

Please choose **only one** of the following:

- ○ Yes
- ○ No

In which program(s)?

Only answer this question if the following conditions are met:
Answer was 'Yes' at question '22 [Cours11]' (Do students in your occupational therapy program have the opportunity to enroll in a universal accessibility course in another program (e.g., architecture) in your university?)

Please write your answer(s) here:

1.
2.
3.
4.
5.

Is it possible for a student to complete an internship in a facility specializing in universal accessibility? (e.g., architectural firm, city department)

Please choose **only one** of the following:

- Yes
- No

Where?

Only answer this question if the following conditions are met:
Answer was 'Yes' at question '24 [Stage1]' (Is it possible for a student to complete an internship in a facility specializing in universal accessibility? (e.g., architectural firm, city department))

Please write your answer here:

In your opinion, what are the deficiencies in teaching universal accessibility in your occupational therapy program (if any)? (Describe in a few words)

Please write your answer here:

Perception of the importance of universal accessibility

In your opinion, on a scale of 0-10, how important is it to address universal accessibility in a professional occupational therapy education program? (10 being the most important and 0 being unimportant)

Please choose the appropriate response for each item:

	1	2	3	4	5	6	7	8	9	10
Is it important to address universal accessibility?	○	○	○	○	○	○	○	○	○	○

Do you have any other information or comments?

Please write your answer here:

Please attach the course syllabus for the accessible course.

Please upload at most one file

Kindly attach the aforementioned documents along with the survey

This will be used for content analysis of course syllabi for universal accessibility courses available in all occupational therapy programs in Canada.

Thank you for your participation!

Submit your survey.
Thank you for completing this survey.
